# Radiation-Aware Path Planning Framework for Mobile Robots in Dynamic Hazardous Environments

**DOI:** 10.3390/s26154937

**Published:** 2026-08-04

**Authors:** Rifatcan Karamanlıoğlu, Nurettin Gökhan Adar, Oğuz Mısır, Davut Ertekin

**Affiliations:** 1 Department of Mechatronics Engineering, Faculty of Engineering and Natural Sciences, Bursa Technical University, 16310 Bursa, Türkiye; 25462877009@ogr.btu.edu.tr (R.K.); gokhan.adar@btu.edu.tr (N.G.A.); oguz.misir@btu.edu.tr (O.M.); 2 Power Electronics Center of Research Laboratories, Electrical and Electronics Engineering Department, Bursa Technical University, 16310 Bursa, Türkiye

**Keywords:** mobile robot, radiation-aware path planning, hazardous environments, A*, chaotic particle swarm optimization, B-Spline smoothing, dynamic obstacle avoidance

## Abstract

This study presents a radiation-aware path planning framework for mobile robots operating in hazardous environments containing radiation sources, shielding structures, and moving obstacles. The proposed method integrates A*-based global planning, chaotic particle swarm optimization (CPSO)-based route refinement, B-Spline trajectory smoothing, and exposure-dependent speed adaptation within a unified dynamic planning architecture. The framework represents the radiation field as a physically parameterized dose-rate map in mSv/h by combining inverse-square source decay with line-of-sight material attenuation through shielding materials. Route generation is therefore evaluated in terms of cumulative absorbed dose, path length, mission time, trajectory roughness, computational cost, success rate, and dynamic obstacle interaction. The proposed method is compared with Pure A*, Informed RRT*, A*-PSO, A*-CPSO, and a risk-aware A*+DWA baseline under identical seed sets and computational budgets. In static scenarios, the proposed method reduced cumulative absorbed dose by approximately 33.3%, 47.2%, and 21.4% compared with Pure A* under low-, medium-, and high-risk conditions, respectively. In dynamic scenarios, the corresponding dose reductions were approximately 32.5%, 41.4%, and 40.1%. Additional ablation, sensitivity, statistical significance, and latency analyses were conducted to isolate the contribution of each component and evaluate computational feasibility. The results show that the proposed framework provides a balanced trade-off between absorbed dose reduction, trajectory feasibility, mission time, and online replanning performance under the tested simulation conditions.

## 1. Introduction

Path planning is a fundamental problem in autonomous mobile robotics, requiring a robot to move from an initial position to a target location through a feasible, collision-free, and task-compatible trajectory. In conventional applications, path quality is commonly evaluated using path length, obstacle avoidance, travel time, smoothness, and computational efficiency [1,2]. However, in hazardous environments such as nuclear facilities, radiological monitoring areas, disaster zones, and sites where direct human access is unsafe, path planning becomes a more complex risk-aware decision-making problem. In such environments, the robot must not only avoid physical obstacles but also account for environmental hazards that may affect mission success, robot survivability, and operational safety [3,4,5].

Radiation-aware navigation is a representative example of this challenge. In environments containing ionizing radiation sources, route safety cannot be evaluated solely by geometric distance or collision-free motion. The total risk depends on both the spatial path followed by the robot and the time spent in regions with elevated radiation intensity [6,7,8,9]. Therefore, a geometrically short and collision-free path may still be undesirable if it passes close to radiation sources or keeps the robot in high-risk regions for a long duration. For this reason, radiation-aware path planning should jointly consider radiation distribution, distance-dependent attenuation, cumulative absorbed dose, obstacle interactions, trajectory smoothness, and velocity selection [6,9,10,11,12,13].

Mobile robot path planning methods have generally developed around deterministic graph search, sampling-based planning, metaheuristic optimization, learning-based decision making, and hybrid planning frameworks [14,15]. Deterministic methods such as A* and Dijkstra provide computationally clear and feasible solutions in structured maps, but they are mostly driven by geometric costs and obstacle constraints, which limits their ability to represent continuous physical risk fields and time-dependent exposure accumulation [10,14,15]. Sampling-based planners such as RRT* offer flexibility in complex search spaces, but their paths often require additional refinement for smoothness, dose accumulation, and execution feasibility. Metaheuristic approaches, including particle swarm optimization, genetic algorithms, and ant colony optimization, can handle multi-objective planning criteria such as path length, safety, and smoothness [1,16,17,18,19]. Nevertheless, these methods may be sensitive to initial solutions, parameter settings, premature convergence, and computational cost, particularly in dynamic and safety-critical environments.

Learning-based and deep reinforcement learning methods have recently attracted attention for adaptive path planning and dynamic obstacle avoidance in unknown or partially known environments [20,21,22,23]. These methods can learn navigation policies through interaction with the environment and may perform well in complex dynamic scenarios. However, in radiation-related missions, relying solely on learned policies may be insufficient because the planning process should remain physically interpretable and safety-oriented. Variables such as radiation intensity, cumulative absorbed dose, obstacle clearance, trajectory smoothness, and velocity constraints should be explicitly represented in the decision process. Thus, radiation-aware planning requires not only adaptability but also physically meaningful cost functions and controllable optimization components.

Hybrid path planning methods aim to overcome the limitations of individual algorithms by combining global planning, local avoidance, route optimization, and trajectory smoothing [12,24,25]. A*-based global planners have been integrated with local planners such as DWA, TEB, and other dynamic obstacle avoidance strategies to improve both global consistency and local feasibility [4,5,12,24,25]. Similarly, hybrid A*–PSO structures have been used to reduce sharp turns, redundant nodes, and the limited refinement capability of standard A* paths through swarm-based optimization [1,18,26]. However, most existing hybrid studies represent environmental risk through distance-based penalties, static cost layers, or predefined safety zones. This representation is limited for radiation environments because radiation is not simply an obstacle or forbidden region; it is a continuous physical risk field whose effect depends jointly on position and exposure time.

Despite recent progress, an important gap remains between radiation-informed navigation and hybrid mobile robot path planning. Existing radiation-related robotic studies mainly focus on radiation mapping, source search, layered costmap-based avoidance, or radiation-informed navigation [6,7,8,9], whereas general mobile robot planning studies mainly address obstacle avoidance, shortest path generation, path smoothness, and dynamic local planning through A*-based, DWA-based, or hybrid global-local strategies [12,24,25]. However, limited attention has been paid to a unified planning framework in which a physically parameterized radiation dose-rate field is propagated consistently through global search, metaheuristic route refinement, trajectory smoothing, velocity adaptation, and dynamic replanning. In particular, the coupled effects of route geometry, absorbed dose, shielding-aware radiation attenuation, mission time, moving-obstacle uncertainty, and velocity selection have not been sufficiently investigated in radiation-aware mobile robot path planning.

To address this gap, this study proposes a hybrid radiation-aware path planning framework for autonomous mobile robots operating in hazardous dynamic environments. The proposed framework formulates path planning not only as a feasible route generation problem, but also as a risk-aware decision problem that jointly considers cumulative absorbed dose, exposure duration, shielding-aware radiation attenuation, dynamic obstacle interactions, and trajectory feasibility. In the proposed approach, an A*-based global planner generates an initial route using a dose-aware multi-objective cost structure. A chaotic particle swarm optimization strategy then refines this route toward lower-dose and geometrically improved regions. B-Spline interpolation converts the optimized route into a smoother and more trackable trajectory [25,26,27,28]. Finally, an exposure-dependent dynamic speed adaptation mechanism adjusts the robot velocity according to local dose-rate intensity to reduce residence time in high-dose-rate regions while avoiding unnecessary mission-time increases in low-risk areas.

To position the proposed framework within the broader state of the art, several additional path-planning paradigms should also be considered, including neural-field, physics-inspired, deformation-aware, population-based, and dynamically constrained trajectory-planning approaches. Zhong et al. formulated optimal robot path planning through a cellular neural network in which target activity propagates through local neural interactions to generate collision-free paths in dynamic environments [29]. Hills and Zhong developed a related thermal-field formulation that treats the target as a heat source and obstacles as boundary conditions, while a cellular neural network enables real-time modeling of the heat-conduction process [30]. Bahwini et al. addressed needle path planning in deformable soft tissue using a bio-heat-transfer-based potential field and finite-element modeling of tissue deformation [31]. For multi-constraint three-dimensional UAV planning, Zhou et al. proposed a crossover-recombination-based global-best brain storm optimization algorithm that uses cubic B-Spline path representation to consider safety, economy, flyability, and trajectory continuity [32]. More recently, Long et al. developed a reinforcement learning-based safe planner that combines multiple candidate paths with Monte Carlo-based human-risk prediction for mobile robots operating around stochastically moving humans [33]. Luo et al. proposed the OTPWR method, which combines globally optimal grid-based path search with differential-flatness-based polynomial trajectory optimization to generate smooth trajectories satisfying wheeled-robot dynamic constraints [34].

These advanced approaches demonstrate that path planning can be formulated through distributed neural dynamics, physical-field analogies, deformable-environment models, population-based global optimization, risk-aware learning, and dynamically constrained trajectory generation. However, their primary objectives and hazard representations differ from those of the present study. The cellular neural network and thermal field methods construct navigation fields through neural or heat-conduction analogies [29,30]; the soft-tissue method focuses on deformation-aware needle insertion [31]; the GBSO-CR method addresses population-based, multi-constraint three-dimensional UAV path planning [32]; the reinforcement learning method models stochastic human-related collision risk [33]; and OTPWR primarily targets global optimality and dynamic feasibility for wheeled-robot trajectories [34]. In contrast, the present study addresses mobile-robot navigation in dynamically occupied radiological environments, where the hazard is represented by a physically parameterized and shielding-aware radiation dose-rate field and where route safety depends jointly on path geometry and time-integrated exposure. Accordingly, the proposed framework is positioned as a radiation-specific hybrid architecture that consistently couples dose-aware global search, bounded event-triggered CPSO refinement, safety-validated B-Spline smoothing, moving-obstacle uncertainty, and exposure-dependent speed adaptation, rather than as a general replacement for these broader planning paradigms.

The novelty of the proposed framework is therefore not claimed to be the individual use of A*, CPSO, B-Spline interpolation, or velocity adaptation. These are established components. The contribution lies in how these components are coupled for radiation-aware dynamic navigation: the same dose-rate field is used in global route generation, local CPSO refinement, cumulative dose evaluation, and speed adaptation; the CPSO stage is restricted to a bounded look-ahead window for online use, the smoothed trajectory is revalidated against safety constraints before execution, and the dynamic planner is evaluated against stronger baselines, including Informed RRT* and a risk-aware A*+DWA hybrid. This coupling allows the method to analyze the trade-off between absorbed dose, path length, trajectory roughness, mission time, success rate, and computational latency within a single reproducible simulation framework.

The main contributions of this study are summarized as follows:

- A physically parameterized radiation dose-rate model is incorporated into the planning problem. The radiation field is expressed in mSv/h using inverse-square decay and line-of-sight material attenuation, instead of being treated only as a normalized abstract risk map.
- A hybrid radiation-aware planning architecture is developed by coupling A*-based global planning, CPSO-based local route refinement, safety-validated B-Spline smoothing, and exposure-dependent speed adaptation under a common dose-aware cost structure.
- The dynamic version of the framework uses a bounded look-ahead CPSO refinement strategy and a two-rate non-blocking simulation structure, allowing for fast reactive replanning while the optimization and smoothing stages improve upcoming trajectory segments in the background.
- The evaluation is strengthened by comparing the proposed framework with Pure A*, Informed RRT*, A*-PSO, A*-CPSO, and a risk-aware A*+DWA baseline under identical seeds and computational budgets.
- The study reports not only mean performance metrics but also ablation results, sensitivity analysis, statistical significance tests, success rates, failure modes, and computational latency, providing a more complete assessment of the proposed method in radiation-aware dynamic navigation.

The remainder of this paper is organized as follows. First, the radiation-aware dynamic path planning problem and the main assumptions are defined. Then, the proposed method is presented. Next, the simulation environment, static and dynamic scenarios, compared methods, and evaluation metrics are described. Finally, the results are discussed in terms of radiation-aware path quality, trajectory feasibility, dynamic obstacle response, and computational performance.

## 2. Problem Formulation

The problem addressed in this study is to generate a collision-free, feasible, smooth, and risk-aware route for a mobile robot operating in a two-dimensional task environment containing radiation sources and non-traversable obstacle regions. In such environments, the risk experienced by the robot depends not only on its distance from radiation sources but also on its location within the physically parameterized dose-rate field and the motion behavior along the route. Therefore, radiation exposure must be considered together with path length, obstacle clearance, and trajectory feasibility during route planning [6,8,9].

The bounded two-dimensional task environment is defined as $E\subset R^{2}$. Non-traversable obstacles or forbidden regions are denoted by O⊂E, and the free space available for robot motion is given by:

(1) $$\begin{array}{cccc} & F=E\setminus O & & \end{array}$$

The start and goal positions are denoted by $q_{s}\in F$ and $q_{g}\in F$, respectively. A route is represented as a curve parameterised over the interval [0,1]:

(2) $$\pi :[0,1]\to F,\pi (0)=q_{s},\pi (1)=q_{g}$$

For a route to be feasible, it must remain in the free space, avoid all non-traversable regions, and maintain a minimum safety distance from obstacles.

The radiation sources in the environment are denoted by $S=\{s_{1},s_{2},\ldots ,s_{N_{s}}\}$. Each source is represented by its position, reference dose rate, anisotropy term, and attenuation characteristic.

The radiation field is represented as a physically parameterized dose-rate field rather than as a normalized abstract risk map. For a robot position x, the local dose rate $\dot{D}\left(x\right)$ is computed in mSv/h by combining inverse-square source decay with line-of-sight material attenuation:

(3) $$\dot{D}\left(x\right)=\sum_{i=1}^{N_{s}}D_{0,i}{\left(\frac{r_{0}}{max\left(r_{i},\left(x\right)r_{min}\right)}\right)}^{2}A_{i}\left(\theta_{i}\right)\exp\left(-\sum_{m\in M}\mu_{m}L_{i,m}\left(x\right)\right)$$

Here, $D_{0,i}$ is the reference dose rate of the i-th source in mSv/h, $r_{i}\left(x\right)$ is the Euclidean distance between the source and the robot, $r_{0}$ is the reference distance, and $r_{min}$ prevents singularity near the source. The term $A_{i}\left(\theta_{i}\right)$ represents source anisotropy and is set to one for isotropic sources. The attenuation term accounts for shielding along the line of sight: $\mu_{m}$ is the material-specific linear attenuation coefficient, and $L_{i,m}\left(x\right)$ is the path length traveled by the line of sight through material m. In this study, air, concrete, steel, and lead attenuation coefficients were used as reported in Table 1. This line-of-sight attenuation term prevents the dose-rate field from passing unchanged through solid shielding regions and allows the local field intensity to decrease according to the material type and the effective shielding thickness.

The risk-aware route planning problem is formulated as a constrained scalar optimization problem:

(4) $$\pi^{*}=\arg\underset{\pi }{min}J\left(\pi \right),\;J\left(\pi \right)=\alpha C_{L}\left(\pi \right)+\beta C_{R}\left(\pi \right)+\gamma C_{S}\left(\pi \right)+\delta C_{O}\left(\pi \right)\;$$

This optimization is subject to the feasibility constraints:

(5) $$\begin{array}{cccc} & \pi (u)\in F,d(\pi (u),O)\geq d_{min},\forall u\in [0,1] & & \end{array}$$

Here, d(π(u),O) denotes the Euclidean distance between a point on the route and the nearest obstacle region, while $d_{min}$ is the required minimum safety distance. In (4), $C_{L}(\pi )$, $C_{R}(\pi )$, $C_{S}(\pi )$, and $C_{O}(\pi )$ represent the path length, the cumulative-dose cost, smoothness cost, and obstacle/safety cost, respectively. The coefficients α, β, γ, and δ define the relative contribution of these terms.

To account for uncertainty in dynamic obstacle prediction and narrow-passage traversal, the obstacle/safety cost $C_{O}\left(\pi \right)$ also includes a variance-aware component. In this study, the safety cost is decomposed as $C_{O}\left(\pi \right)=C_{occ}\left(\pi \right)+\lambda_{\sigma }C_{\sigma }\left(\pi \right)+\lambda_{n}C_{narrow}\left(\pi \right)$, where $C_{occ}\left(\pi \right)$ penalizes proximity to occupied or non-traversable cells, $C_{\sigma }\left(\pi \right)$ penalizes traversal through regions with high predicted occupancy variance, and $C_{narrow}\left(\pi \right)$ penalizes geometrically narrow passages in which small localization, sensing, or tracking errors may cause collision. The coefficients $\lambda_{\sigma }$ and $\lambda_{n}$ control the contributions of the predicted-uncertainty and narrow-passage terms, respectively. This variance-aware safety cost discourages paths that pass through tight bottlenecks or uncertain future obstacle regions, where small sensing, localization, or tracking errors could produce collision or an unintended increase in accumulated dose.

The objective of this formulation is not only to minimize path length. A geometrically short route may pass through high-risk regions and produce high cumulative absorbed dose. Conversely, excessive radiation avoidance may result in a long, highly curved, or difficult-to-track trajectory. Therefore, the proposed formulation aims to balance path length, cumulative absorbed dose, trajectory smoothness, and obstacle clearance. The reported Total Dose (mSv) values should be interpreted as physically parameterized simulation doses rather than measurements from a specific nuclear facility.

## 3. Methodology

The proposed method solves the radiation-aware route planning problem through a four-stage structure. First, an A*-based global planner generates a feasible and risk-informed initial route. Second, CPSO-based route refinement improves this route according to a weighted composite cost function. Third, B-Spline smoothing converts the optimized discrete route into a continuous trajectory. Finally, dynamic speed adaptation adjusts the velocity profile by considering local radiation risk, trajectory curvature, and obstacle proximity. The overall workflow is expressed as

(6) $$\pi_{A^{*}}\overset{\mathrm{CPSO}}{\to }\pi_{\mathrm{CPSO}}\overset{B\text{-}\mathrm{Spline}}{\to }\tau (u)\overset{\mathrm{Dynamic}\;\mathrm{Speed}\;\mathrm{Adaptation}}{\to }v(u)$$

Here, $\pi_{A^{*}}$ is the initial route generated by A*, $\pi_{\mathrm{CPSO}}$ is the refined discrete route, τ(u) is the continuous B-Spline trajectory, and v(u) is the final velocity profile.

### 3.1. A*-Based Global Initial Route

In the first stage, the two-dimensional task environment is represented as a grid map consisting of free and non-traversable cells. Each grid cell is treated as a graph node, and A* is used to generate an initial route between $q_{s}$ and $q_{g}$. The purpose of this stage is not to obtain the final optimum solution, but to provide a collision-free and task-feasible initial route for the subsequent CPSO-based refinement.

The transition cost between neighboring nodes n and $n^{\prime }$ is defined as

(7) $$c(n,n^{\prime })=w_{d}c_{d}(n,n^{\prime })+w_{r}c_{r}(n,n^{\prime })+w_{o}c_{o}(n,n^{\prime })$$

Here, $c_{dist}$ is the geometric transition cost, $c_{dose}$ is the dose-aware transition cost associated with the average local dose rate along the transition, and $c_{safe}$ is the safety cost associated with proximity to non-traversable regions. The weights $w_{d}$, $w_{r}$, and $w_{o}$ determine the relative importance of these components. Thus, A* is guided not only toward shorter transitions but also toward safer and lower-risk regions.

### 3.2. CPSO-Based Route Refinement

Although the A*-generated route $\pi_{A^{*}}$ is feasible, it may contain sharp turns, redundant deviations, or segments that can be improved in terms of radiation exposure. Therefore, CPSO-based refinement is applied to improve the route geometry while keeping the start and goal positions fixed.

To balance cost components with different physical scales, each raw cost component is normalized with respect to the initial A* route:

(8) $$\begin{array}{cccc} & {\bar{C}}_{m}(\pi_{i})=\frac{C_{m}(\pi_{i})}{C_{m}(\pi_{A^{*}})+\epsilon_{\mathrm{norm}}},m\in \{L,R,S,O\} & & \end{array}$$

Here, $C_{m}(\pi_{i})$ is the raw cost component of the candidate route $\pi_{i}$, and ${\bar{C}}_{m}(\pi_{i})$ is the corresponding normalized cost. The index m∈{L,R,S,O} denotes path length, dose-related cost, smoothness, and obstacle/safety cost. The term $\epsilon_{\mathrm{norm}}$ prevents division by zero.

The CPSO fitness function is defined as

(9) $$\begin{array}{cccc} & F(\pi_{i})=\sum_{m\in \{L,R,S,O\}}w_{m}{\bar{C}}_{m}(\pi_{i})+\lambda P(\pi_{i}) & & \end{array}$$

The penalty term $P(\pi_{i})$ is activated only when a collision, forbidden-region violation, or minimum-distance violation occurs. The parameter λ controls the effect of constraint violation on the fitness value.

Chaotic search behavior is included to improve search diversity and reduce premature convergence. The chaotic sequence is used to update the inertia coefficient as

(10) $$z_{t+1}=\eta z_{t}(1-z_{t}),\;\begin{array}{cccc} & \omega_{t}=\omega_{min}+(\omega_{max}-\omega_{min})z_{t} & & \end{array}$$

Here, $z_{t}$ is the chaotic variable at iteration t, η is the logistic map parameter, and $\omega_{t}$ is the iteration-dependent inertia coefficient. The bounds of the inertia coefficient are denoted by $\omega_{min}$ and $\omega_{max}$. The chaotic map parameter is kept constant in all experiments and reported in the simulation parameter table.

In dynamic execution, CPSO is not applied to the entire remaining route at every control step. Instead, a bounded rolling-window refinement is used. The local optimization window is defined over the upcoming route segment within a fixed look-ahead distance from the current robot position. Only the waypoints inside this local window are treated as optimization variables, while the already executed trajectory and the distant global route remain fixed. This design reduces the dimensionality of the CPSO search space and allows metaheuristic refinement to improve upcoming trajectory segments without blocking high-frequency reactive replanning.

### 3.3. B-Spline-Based Trajectory Smoothing

The refined route $\pi_{\mathrm{CPSO}}$ may still contain abrupt directional changes due to its discrete and grid-based structure. Therefore, B-Spline smoothing is used to convert the discrete route into a continuous and more trackable trajectory. The smoothed trajectory is defined as

(11) $$\begin{array}{cccc} & \tau (u)=\sum_{j=0}^{n}N_{j,r}(u)p_{j},u\in [0,1] & & \end{array}$$

Here, $p_{j}$ denotes the B-Spline control points, and $N_{j,r}(u)$ represents the B-Spline basis functions of degree r. In this study, a cubic clamped B-Spline is used to preserve the start and goal positions.

After smoothing, the continuous trajectory is resampled and checked for free-space validity, obstacle collision, and minimum safety distance. If an invalid sample point is detected, the smoothing configuration is adjusted conservatively. If no valid smoothed trajectory can be obtained, the collision-free discrete route is used as a fallback. This mechanism prevents smoothness improvement from being achieved at the expense of safety.

### 3.4. Dynamic Speed Adaptation

In the final stage, dynamic speed adaptation is applied along the smoothed trajectory. The goal is to reduce the residence time in high-dose-rate regions while preserving safe motion in high-curvature areas and near obstacles. Therefore, the velocity is not determined only by the local dose rate; it is also constrained by curvature and obstacle proximity. The velocity profile is defined as

(12) $$v\left(u\right)=clip\left(v_{0},+,\alpha_{D}\hat{\dot{D}}\left(u\right)-\alpha_{\kappa }\hat{\kappa }\left(u\right)-\alpha_{d}{\hat{c}}_{d}\left(u\right)v_{min}v_{max}\right)$$

Here, $v_{0}$ is the nominal cruising speed, $v_{min}$ and $v_{max}$ are the velocity limits, $\hat{\dot{D}}\left(u\right)$ is the normalized local dose-rate term derived from $\dot{D}\left(x\right)$, $\hat{\kappa }\left(u\right)$ is the normalized trajectory curvature, and ${\hat{c}}_{d}\left(u\right)$ is the obstacle proximity penalty. The coefficients $\alpha_{D}$, $\alpha_{\kappa }$, and $\alpha_{d}$ determine the relative influence of these factors. The $clip\left(\cdot \right)$ operator ensures that the velocity remains within the allowed limits.

This structure allows for controlled acceleration in high-dose-rate regions while reducing velocity in sharp turns and narrow passages. Therefore, the method does not produce uncontrolled acceleration in hazardous regions; any speed increase is constrained by curvature, obstacle proximity, acceleration limits, and predefined velocity bounds.

After speed adaptation, the cumulative absorbed dose is computed by integrating the local dose rate over the travel time:

(13) $$D_{tot}=\int_{0}^{T}\frac{\dot{D}\left(x\left(t\right)\right)}{3600}\;dt=\int_{0}^{1}\frac{\dot{D}\left(\gamma \left(s\right)\right)}{3600}\frac{∥\gamma^{\prime }\left(s\right)∥}{v\left(s\right)}\;ds$$

where $D_{tot}$ is the cumulative absorbed dose in mSv, $\dot{D}\left(\gamma \left(s\right)\right)$ is the local dose rate in mSv/h along the trajectory, and $v\left(s\right)$ is the speed profile in m/s. The factor 1/3600 converts the dose-rate unit from mSv/h to mSv/s because the travel time is computed in seconds. This formulation accounts for both the spatial dose-rate distribution and the time spent along each trajectory segment.

## 4. Simulation Design

This section presents the simulation design used to evaluate the performance of the proposed radiation-aware path planning architecture in static and dynamic task environments. The experimental setup was designed to jointly analyze the reduction in Total Dose (mSv), preservation of route feasibility, improvement of trajectory smoothness, and maintenance of stable navigation behavior under dynamic obstacles.

Therefore, the evaluation was not limited to path length or goal-reaching success. Instead, Total Dose (mSv), mission time, trajectory smoothness, computational cost, and convergence behavior were considered together. In this way, the simulation design evaluates not only the ability of the proposed method to generate short routes, but also its ability to produce risk-aware and operationally feasible trajectories.

All simulations were conducted in an 80×80 grid-based workspace. Each cell was represented as either a traversable area or an obstacle region in a binary occupancy map. This resolution was selected because it allows narrow passages, clustered radiation fields, and dynamic obstacle interactions to be represented while keeping the computational cost manageable during multiple replanning operations.

The radiation field was modeled as a physically parameterized dose-rate field in mSv/h using inverse-square source decay and line-of-sight material attenuation. Radiation was therefore not treated as a hard forbidden region or a normalized abstract risk layer, but as a continuous dose-rate field affecting both route cost and cumulative absorbed dose. The source dose-rate ranges and shielding materials used in the simulations are summarized in Table 2.

The experimental setup consists of two main conditions: a static environment and a dynamic environment. In the static environment, the obstacle layout and radiation dose-rate field remained fixed throughout the planning process. This condition allowed the compared methods to be evaluated in terms of route quality, cumulative absorbed dose reduction, trajectory smoothness, mission time, and planning cost under fixed dose-rate fields.

**Figure 1:**
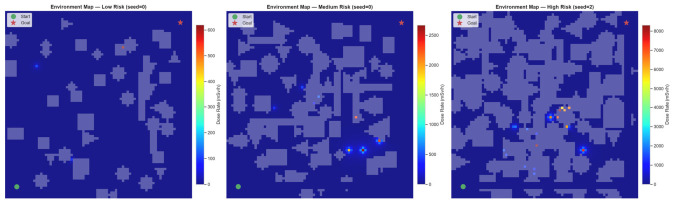
Static simulation environments under three risk levels.

**Figure 2:**
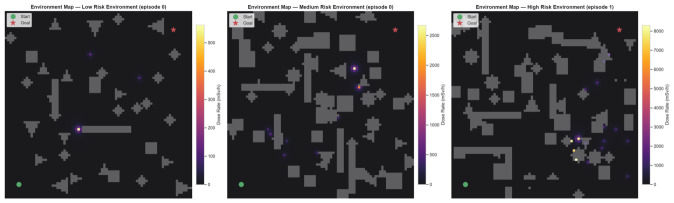
Dynamic simulation environments under three risk levels.

In the dynamic environment, the same risk profiles were preserved, while moving obstacles were added to evaluate replanning, goal convergence, failure modes, and online execution latency under changing environmental conditions. Six methods were evaluated in both static and dynamic experiments: Pure A*, Informed RRT*, A*-PSO, A*-CPSO, risk-aware A*+DWA, and the proposed method. In the static experiments, 100 independent seeds were used for each risk level and each method, resulting in 3×6×100=1800 static runs. In the dynamic experiments, 100 independent episodes were generated for each risk level and each method, resulting in 3×6×100=1800 dynamic episodes. Representative static environment examples are shown in Figure 1, and dynamic environment examples are shown in Figure 2.

Figure 1 presents representative static simulation environments for low-, medium-, and high-risk levels. The gray regions represent non-traversable static obstacles, while the background color distribution represents the dose-rate field. In the color scale, dark red regions indicate high local dose-rate regions, whereas regions transitioning toward blue indicate lower local dose-rate regions. These maps were used to compare the route generation behavior of the methods under different risk intensities and obstacle layouts.

Figure 2 presents representative dynamic simulation environments for low-, medium-, and high-risk levels. In these maps, the background color distribution represents the dose-rate field, where warmer colors indicate higher local dose-rate regions. The gray geometric regions represent non-traversable static obstacles, the green circle denotes the start position of the robot, and the red star denotes the goal position. As the risk level increases in the dynamic environment, both the spatial influence of the radiation sources and the number of moving obstacles increase. This structure was used to evaluate not only the static risk avoidance behavior of the proposed method but also its replanning capabilities under changing obstacle conditions.

### 4.1. Compared Methods

Six methods were compared in the experiments: Pure A*, Informed RRT*, A*-PSO, A*-CPSO, a risk-aware A*+DWA hybrid, and the proposed method. Pure A* was used as the deterministic graph-search baseline. Informed RRT* was used as the sampling-based baseline because it restricts the sampling domain after the first feasible solution and provides a stronger comparison than uninformed sampling-based planning. A*-PSO and A*-CPSO were used to isolate the effects of standard and chaotic swarm-based route refinement. The risk-aware A*+DWA hybrid was used to represent a global-local planning baseline for dynamic obstacle avoidance. The proposed method differs from these baselines by coupling dose-aware A* planning, bounded CPSO refinement, safety-validated B-Spline smoothing, and exposure-dependent speed adaptation within the same dynamic planning pipeline.

These methods were selected to analyze the effect of the components of the proposed architecture in a progressive manner. The difference between Pure A* and A*-PSO indicates the effect of metaheuristic route refinement. The difference between A*-PSO and A*-CPSO evaluates the contribution of chaotic search behavior to the route improvement process. The difference between A*-CPSO and the proposed method reveals the combined effect of B-Spline-based trajectory smoothing and dynamic speed adaptation.

In dynamic scenarios, because moving obstacles create a strong requirement for fast replanning, the PSO/CPSO components were not treated as continuous online optimization procedures. Instead, they were considered event-triggered limited refinement components. Therefore, the dynamic environment results reflect the combined behavior of replanning, trajectory smoothing, and speed adaptation rather than the full online effect of metaheuristic optimization. In particular, when the current route remains valid, or when there is no meaningful deviation between the new and existing routes, it is expected that A*-PSO and A*-CPSO variants may produce results close to or identical to Pure A*. For this reason, the intermediate variants in the dynamic experiments should be interpreted as comparison stages representing limited event-triggered refinement under fast replanning, rather than as full-time online optimization algorithms.

To ensure a fair comparison, all methods were evaluated using identical start–goal pairs, obstacle layouts, radiation dose-rate fields, dynamic-obstacle trajectories, random seeds, and measurement-noise realizations. In addition, the same safety limits, grid resolution, collision-checking frequency, and control period were used for all methods.

Informed RRT* was used as the sampling-based baseline because it provides informed sampling after the first feasible solution. The risk-aware A*+DWA baseline was used to represent a relevant hybrid global-local planning family for dynamic obstacle avoidance. Its global layer uses the same dose-rate and safety cost layers, whereas its local DWA controller evaluates candidate rollouts using heading, clearance, velocity, and radiation-related costs.

### 4.2. Static Environment Experiments

In the static environment experiments, the robot was evaluated in a task environment containing a fixed dose-rate field and fixed obstacles. The start and goal positions were set to (5,5) and (75,75), respectively. Three static risk profiles were used: low-risk, medium-risk, and high-risk environments. These risk levels were differentiated in terms of the number of radiation sources, source intensity, spatial attenuation coefficient, and environmental complexity.

For each risk level and each method, 100 independent random seeds were used. Since six methods were evaluated under three risk levels, a total of 3×6×100=1800 static runs were conducted. In each scenario, all compared methods were evaluated under the same dose-rate distribution, obstacle layout, start–goal configuration, and random seed. Thus, the performance differences were attributed directly to the planning strategy rather than to environmental randomness.

### 4.3. Dynamic Environment Experiments

Dynamic environment experiments were constructed by adding moving obstacles and online replanning components to the static experimental structure. In this environment, the start position of the robot was set to (6,6), and the goal position was set to (72,72). The low-, medium-, and high-risk profiles used in the static experiments were also preserved in the dynamic environment generation. However, the number of moving obstacles was increased according to the risk level. Three moving obstacles were used in the low-risk environment, five in the medium-risk environment, and eight in the high-risk environment. This arrangement ensured that, as the risk level increased, not only radiation exposure but also dynamic obstacle interactions became more challenging.

For each risk level and each method, 100 independent dynamic episodes were generated using paired random seeds. Since six methods and three risk levels were evaluated, the dynamic benchmark contains 3×6×100=1800 dynamic episodes in total. For each episode, all compared methods used the same static obstacle layout, radiation dose-rate field, moving-obstacle trajectories, and measurement-noise realization. This paired design was used to reduce stochastic variability and to support the paired statistical tests reported in Section 5.6.

In each dynamic episode, the positions of the moving obstacles were updated over time. Two behavior models were used for moving obstacles: a follower obstacle and patrol-type obstacles. The follower obstacle was modeled as an active dynamic risk element moving toward the estimated current position of the robot. This obstacle updates its direction at each time step based on the robot position, thereby increasing the replanning requirement. Patrol-type obstacles were modeled as independently moving obstacles with randomly assigned initial velocity components. These obstacles reverse their motion direction when they reach the environment boundaries. Thus, the dynamic scenarios represent both an active robot-following obstacle behavior and transient obstacle interactions caused by independently moving objects.

To avoid assuming perfect deterministic knowledge of moving-obstacle states, a Kalman-based prediction layer was used to estimate short-horizon obstacle positions. The measured obstacle position was corrupted by measurement noise, and the predicted state was propagated over a 3 s horizon using the process- and measurement-noise settings reported in Table 1. The predicted covariance was used to expand the local occupancy influence of each moving obstacle, producing a spatiotemporal risk region rather than a single deterministic obstacle point. Therefore, collision checking and local replanning were based not only on the current obstacle location but also on predicted future occupancy uncertainty.

In the dynamic environment experiments, the occupancy map was updated at each time step according to the current positions of moving obstacles. The robot’s motion decision was dynamically reevaluated by considering the current obstacle positions and the radiation dose-rate field. This structure aims to analyze the goal convergence and safe navigation behavior of the methods not only in static dose-rate fields but also under changing environmental conditions. The collision-checking frequency and the replanning frequency were both set to 10 Hz. An episode was terminated when the robot reached the goal tolerance region, collided with a moving obstacle, collided with a static obstacle, violated the dose or dose-rate constraint, reached the maximum episode length, failed to find a feasible path, encountered planner failure, or stagnated without meaningful progress. All episode outcomes were retained in the raw dynamic result file; failed runs were not removed from success-rate or failure-mode analyses.

To keep the computational cost under control, PSO/CPSO-based full route refinement was not executed at every time step. In dynamic episodes, fast A*-based local replanning was used as the main execution layer against moving-obstacle updates. PSO/CPSO refinement and B-Spline smoothing were activated only when the current route was significantly degraded, when a meaningful deviation occurred between the new route and the existing route, or when additional adjustment was required for trajectory feasibility. Therefore, in dynamic scenarios, the A*-PSO and A*-CPSO variants represent limited event-triggered refinement under fast replanning rather than continuously optimizing methods as in the static experiments. Each local CPSO activation was logged together with its trigger reason, runtime, pre-refinement cost, post-refinement cost, and acceptance status. These logs were used to quantify how often CPSO was activated and whether the additional computation produced a measurable improvement under dynamic conditions.

The dynamic planner was simulated using a two-rate non-blocking execution structure. The reactive A*-based replanning layer operates at the control frequency to handle immediate obstacle updates and maintain a feasible route. At the lower scheduled rate, the local CPSO and B-Spline stages operate on the bounded look-ahead segment as a background refinement layer. A refined segment is accepted only if it improves the local cost and satisfies collision, clearance, and dose-rate constraints; otherwise, the current reactive A* segment is retained. This structure prevents the metaheuristic refinement from blocking immediate obstacle-avoidance decisions.

### 4.4. Simulation Parameters

The main simulation and algorithmic parameters used in the experiments are summarized in Table 1. Table 1 reports the internal settings required for reproducing the results, including A* transition weights, CPSO population size, number of iterations, inertia bounds, acceleration coefficients, penalty coefficient, logistic-map parameter, bounded look-ahead and runtime limits, B-Spline parameters, safety distance, goal tolerance, replanning threshold, collision-checking and replanning frequencies, speed adaptation coefficients, radiation-model parameters, variance-based safety-cost weights, Kalman-tracker parameters, and common computational budgets. A grid-cell size of 1.0 m was assumed; therefore, path length and velocity are reported in meters and meters per second, respectively.

All compared methods were executed under a common computational budget. The maximum planning wall-time was set to 500 ms, the maximum iteration budget was set to 60 where applicable, and the dynamic control period was set to 100 ms. The same safety distance, goal tolerance, replanning threshold, collision-checking frequency, and replanning frequency were used across all methods. The stopping criteria were also defined consistently: Pure A^*^ terminates when the goal heuristic is satisfied or when the open set is exhausted, Informed RRT^*^ terminates when the common wall-time budget is reached and returns the best solution found, A*-PSO and A*-CPSO use the same A* termination rule followed by the fixed swarm-iteration budget, the risk-aware A*+DWA baseline uses the same A* global-planning termination rule and terminates the DWA rollout when the goal tolerance or the common iteration budget is reached, and the proposed method uses the same A* and CPSO budgets followed by safety-validated B-Spline smoothing and speed adaptation.

### 4.5. Risk Profiles

Three different risk profiles were used in the static and dynamic environments: low risk, medium risk, and high risk. These profiles were designed to gradually increase the spatial intensity and exposure complexity of the radiation dose-rate field. The risk levels were mainly differentiated by the number of radiation sources, source dose-rate range, shielding materials, and environmental complexity.

The low-risk environment represents a relatively easier navigation condition due to lower source density and faster radiation attenuation. The medium-risk environment represents an intermediate difficulty level that allows the methods to be compared under balanced environmental conditions. The high-risk environment contains a larger number of stronger radiation sources and more complex shielding configurations, producing broader high-dose-rate regions. This structure forces the planning algorithms not only to generate short routes but also to identify safer passage regions that reduce prolonged dose accumulation.

The source dose-rate ranges define the reference source strengths used in the physically parameterized simulation field. The shielding materials and attenuation coefficients are used in the line-of-sight attenuation term of Equation (3). These values provide an mSv/h-scaled simulation environment for comparative evaluation. However, deployment in a real nuclear facility would require site-specific source characterization, detector calibration, and map validation.

The source numbers indicate the actual generation ranges defined in the simulation code. Radiation sources were generated with a clustered distribution structure. In each scenario, the total number of sources was determined according to the corresponding risk profile; sources were distributed around several clusters, and the first cluster was represented with a higher weight. The source intensity values and attenuation coefficients are simulation parameters used to generate different risk intensities; they should not be interpreted as direct physical field calibration values.

This structure creates a more challenging test environment in which the robot must not only generate a geometrically short route but also balance risk, distance, mission time, and trajectory feasibility under radiation fields with different intensities.

### 4.6. Evaluation Metrics

The performance of the proposed radiation-aware dynamic path planning framework was analyzed using seven main metrics covering safety, efficiency, feasibility, and operational stability. These metrics were selected to evaluate not only the ability of the method to generate a geometric route but also its ability to reduce radiation risk and maintain mission continuity under dynamic environmental conditions. For all metrics except success rate, lower values indicate safer and more efficient solutions. The performance metrics used in the experimental evaluation are presented in Table 3.

Total Dose (mSv) and success rate were used as the main indicators of safety and mission success. Total Dose represents the cumulative absorbed dose obtained by integrating the local dose rate along the executed trajectory over travel time. It is reported in mSv and is used as the primary radiation-related comparison metric among the planning methods.

Success rate and convergence should be interpreted together. In this study, a mission was considered successful when the robot reached the predefined goal tolerance region without collision or safety violation. Therefore, the convergence metric does not need to be zero; it indicates the remaining Euclidean distance between the robot and the goal center at the end of the episode. Thus, the success rate represents goal-region reaching reliability, while the convergence metric provides complementary information about the final distance to the goal center.

Operational efficiency and trajectory feasibility were evaluated through path length, mission time, roughness, and computational cost. The path length and time metrics reflect geometric route efficiency and total travel duration according to the velocity profile. The suitability of the trajectory for mobile robot kinematics was evaluated using the roughness metric, which measures the average absolute heading change between consecutive trajectory segments in radians. The real-time applicability of the method was analyzed using CPU time, which represents the computational cost required for planning and route generation. Since high variance in computational cost is expected in dynamic scenarios due to replanning requirements and obstacle interactions, all results are reported as mean ± standard deviation for each method.

### 4.7. Radiation Model Validation

**Figure 3:**
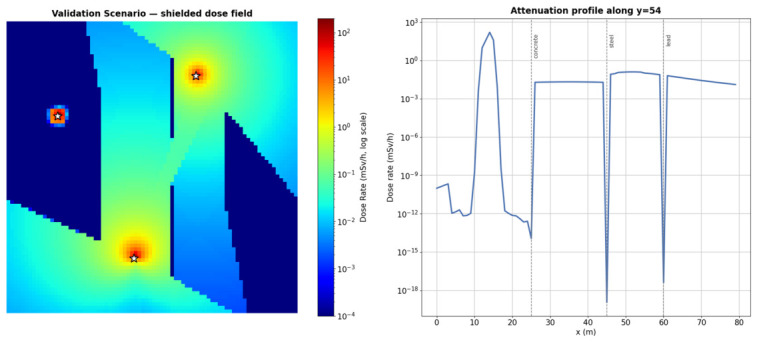
Radiation-model verification and uncertainty analysis scenario. (**Left**) Log-scaled dose-rate field (mSv/h) with concrete, steel and lead shielding walls and one directional source. (**Right**) Dose-rate attenuation profile along the source row, showing the exponential decay behind each wall consistent with exp(−mu*L). White asterisks indicate radiation-source locations.

To substantiate the physical radiation model beyond the comparative benchmarks, a dedicated validation scenario was constructed. It contains three shielding walls of different materials, namely concrete, steel and lead, placed between the sources and the traversable corridor, together with one directional (anisotropic) source. Detector uncertainty is modeled as multiplicative log-normal measurement noise with a 15% standard deviation, and map uncertainty as a spatially smoothed perturbation of +/−20% amplitude. For each realization, the route is planned on the noisy or perturbed dose-rate map, whereas the accumulated dose is always evaluated on the true field, which isolates the effect of imperfect radiation information on the planning outcome. The framework can additionally ingest externally supplied dose-rate maps through a dedicated interface, enabling future evaluation on measured or publicly available benchmark fields. As shown in Figure 3, the dose rate drops sharply behind each shielding wall in agreement with the exponential exp(−mu*L) attenuation law, and the strongest attenuation is obtained behind the lead wall, consistent with its higher linear attenuation coefficient.

## 5. Results and Discussion

This section presents the performance of the proposed radiation-aware path planning framework under static and dynamic environmental conditions. The results are evaluated in terms of Total Dose (mSv), path length, mission time, trajectory roughness, computational cost, convergence behavior, and success rate. The reported Total Dose values correspond to physically parameterized simulation doses computed from the mSv/h dose-rate field and the executed velocity profile. Therefore, these values are used for comparative evaluation among planning methods and should not be interpreted as direct measurements from a specific nuclear facility.

### 5.1. Static Environment Results

The static experiments were conducted under fixed obstacle configurations and fixed radiation dose-rate fields. Table 4, Table 5 and Table 6 summarize the results for low-, medium-, and high-risk scenarios.

**Figure 4:**
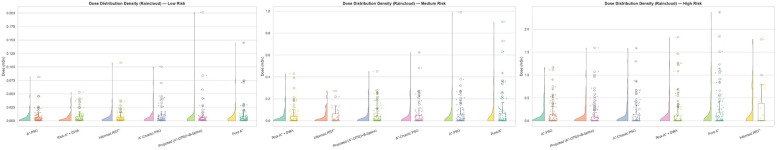
Cumulative absorbed dose distributions in the static environment experiments.

**Figure 5:**
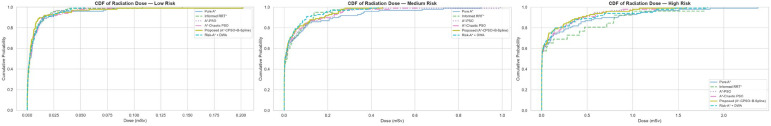
CDFs of cumulative absorbed dose distributions in the static environment experiments.

Figure 4 and Figure 5 present the distribution-based behavior of Total Dose (mSv) in the static scenarios. The raincloud plots and CDF curves generally show lower-dose distributions for the proposed method relative to Pure A* and Informed RRT*, with the clearest separation in the medium-risk scenario. In the high-risk scenario, however, some PSO-based and DWA-based baselines achieve lower mean dose values than the proposed method; therefore, the distribution plots should be interpreted together with Table 4, Table 5 and Table 6.

**Figure 6:**
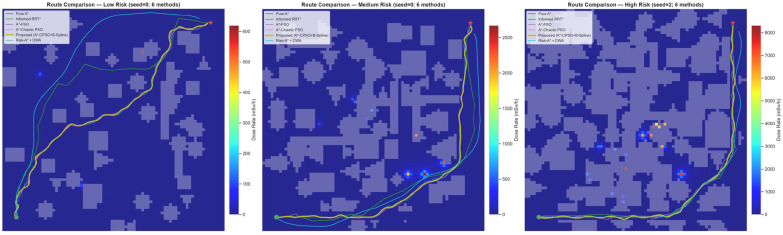
Trajectory comparisons in static environments.

Figure 6 compares representative trajectories generated by the methods. The proposed method produces smoother and more controlled trajectory profiles, while the representative route examples illustrate how dose-aware refinement and smoothing alter the geometric path. These examples are illustrative rather than a substitute for the aggregate comparison in Table 4, Table 5 and Table 6, in which the proposed method achieved the shortest mean path at all three static risk levels.

**Figure 7:**
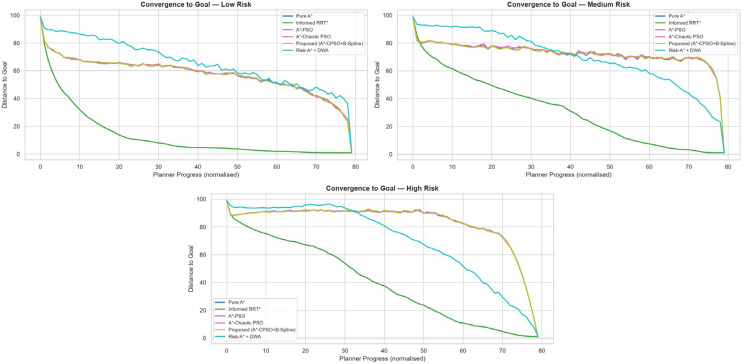
Convergence-to-goal behavior in static environment experiments.

Figure 7 shows the convergence behavior in static environments. All methods generally reached the goal region, indicating that the dose reduction achieved by the proposed method did not compromise goal convergence.

**Figure 8:**
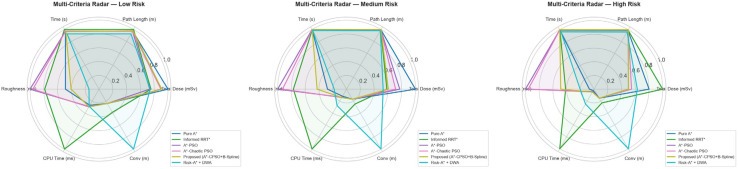
Multi-criteria radar chart comparisons in static environments.

Figure 8 summarizes the multi-criteria performance trade-off. The radar charts confirm that the proposed method should not be evaluated as a method that is absolutely superior in every single metric. Instead, its main advantage is the more balanced trade-off between Total Dose (mSv), trajectory smoothness, mission time, path length, and computational cost.

As expected, Total Dose (mSv) increased with the environmental risk level. Based on the rounded mean values reported in Table 4, Table 5 and Table 6, the proposed method produced Total Dose values of 0.006, 0.038, and 0.158 mSv under low-, medium-, and high-risk conditions, respectively. Relative to Pure A*, these values correspond to approximate reductions of 33.3%, 47.2%, and 21.4%. Therefore, the proposed framework reduced cumulative dose relative to Pure A* across all three static risk levels, although it did not achieve the lowest dose among all baselines in every scenario.

The detailed metric comparison reveals different performance patterns across risk levels. In the low-risk scenario, the proposed method shared one of the lowest displayed mean Total Dose values and achieved the shortest path, shortest mission time, and lowest trajectory roughness. In the medium-risk scenario, the proposed method achieved the lowest mean Total Dose, path length, mission time, and trajectory roughness. In the high-risk scenario, A*-PSO and risk-aware A*+DWA achieved the lowest displayed mean Total Dose of 0.126 mSv, whereas the proposed method achieved the shortest path and mission time. Pure A* produced the lowest trajectory roughness and planning time in the high-risk scenario.

The roughness results show that the proposed method consistently reduced abrupt heading changes compared with the A*-PSO and A*-CPSO variants, supporting the contribution of the safety-validated B-Spline stage. It achieved the lowest mean roughness among all methods in the low- and medium-risk scenarios. In the high-risk scenario, however, Pure A* achieved a lower roughness value than the proposed method. Thus, the proposed framework should be interpreted as providing a balanced multi-metric improvement rather than absolute superiority in every individual metric.

In the updated static experiments, the proposed method did not introduce a path-length or mission-time penalty. Instead, it achieved the shortest mean path and mission time at all three risk levels. Its planning time remained below the common 500 ms computational budget, although it was higher than that of the simpler A*-based variants, particularly in the medium- and high-risk scenarios.

Figure 4 and Figure 5 show that the proposed method generally shifts the Total Dose distribution toward lower values relative to Pure A* and Informed RRT*, particularly in the medium-risk scenario. In the high-risk scenario, although the proposed method reduces dose relative to Pure A* and Informed RRT*, some PSO-based and DWA-based baselines achieve lower mean dose values. Figure 6, Figure 7 and Figure 8 further demonstrate that the proposed method provides favorable combined performance in terms of path length, mission time, and trajectory smoothness. Overall, the static results support a balanced trade-off rather than uniform dominance across every metric and baseline.

### 5.2. Dynamic Environment Results

The dynamic experiments evaluated the methods under moving obstacles and online replanning requirements. Table 7, Table 8 and Table 9 summarize the results for low-, medium-, and high-risk dynamic scenarios.

To further clarify the robustness of the dynamic evaluation, the episode outcomes were also analyzed across all methods and risk levels. The dynamic benchmark contained 1800 episodes in total. Among these episodes, 1615 reached the goal, 127 ended with collision with a moving obstacle, and 58 ended with no feasible path being found. No failed episode was removed from the raw dynamic dataset.

For the proposed method, the success rates were 98%, 89%, and 89% under low-, medium-, and high-risk conditions, respectively. This trend is consistent with the increasing number of moving obstacles and the stronger dose-rate gradients in the medium- and high-risk scenarios.

The updated dynamic results show that the proposed method maintains reliable navigation performance while reducing cumulative absorbed dose under moving-obstacle conditions. The reduction is more clearly supported by the paired statistical analysis reported in Section 5.6 because the raw dynamic tables include large stochastic variations caused by obstacle motion and replanning events. Therefore, the dynamic comparison should be interpreted together with the paired confidence intervals and effect sizes rather than only through unpaired mean ± standard deviation values.

The A*-PSO and A*-CPSO variants produced values close to Pure A* in several dynamic metrics. This behavior is expected because, in the dynamic setting, PSO/CPSO refinements were activated as limited event-triggered components rather than continuous online optimizers. When the current route remained valid, fast A*-based replanning dominated the execution process. Therefore, the dynamic results of these intermediate methods should be interpreted as limited refinement behavior under fast replanning rather than full-time metaheuristic optimization. The specific contribution of local CPSO is quantified separately in Section 5.5.

**Figure 9:**
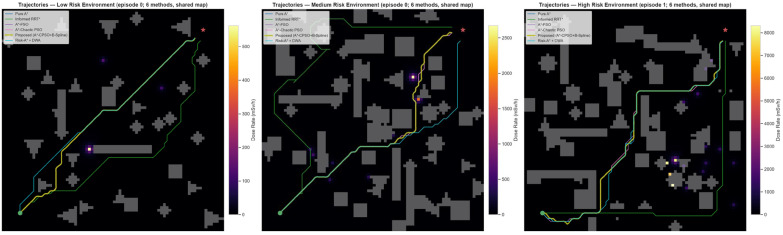
Trajectory comparisons in dynamic environment experiments.

**Figure 10:**
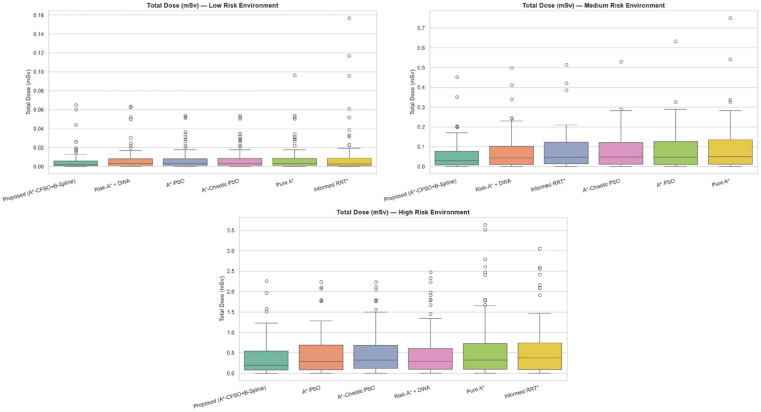
Total Dose (mSv) distributions in dynamic environment experiments.

**Figure 11:**
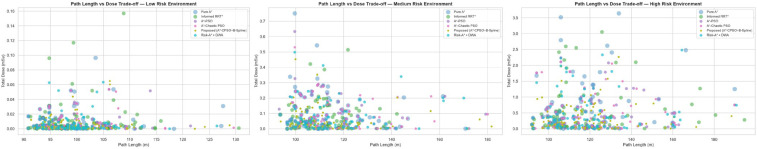
Path length–dose trade-off in dynamic environment experiments.

**Figure 12:**
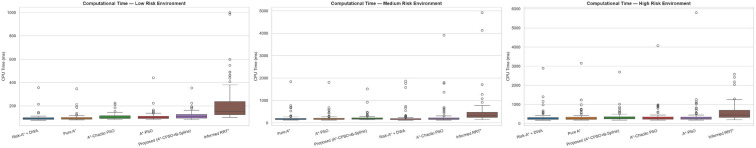
Step-latency distributions in dynamic environment experiments.

**Figure 13:**
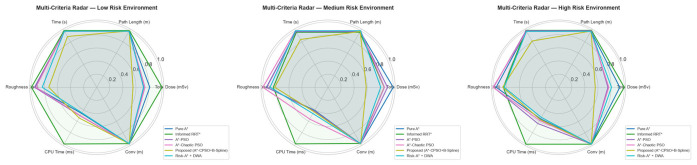
Multi-criteria radar chart comparisons in dynamic environment experiments.

Figure 9, Figure 10, Figure 11, Figure 12 and Figure 13 summarize the dynamic trajectory, dose, path-length, computational, and multi-criteria behavior of the compared methods. Figure 9 illustrates trajectory behavior under moving-obstacle conditions. Figure 10 shows the Total Dose distributions, and Figure 11 shows the path length–dose trade-off. Figure 12 summarizes the step-latency distributions, while Figure 13 presents the multi-criteria radar comparison. Overall, the dynamic results demonstrate that the proposed method provides stable dose-aware navigation under moving obstacles.

### 5.3. Ablation Study

**Figure 14:**
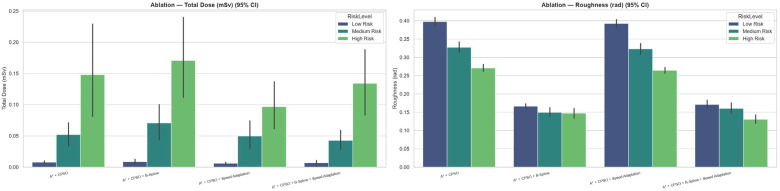
Component-wise contribution to accumulated dose (**left**) and trajectory roughness (**right**) in the ablation study across risk levels.

To attribute the observed gains to individual components, four variants of the proposed pipeline were evaluated using an independent ablation benchmark consisting of 100 seeds per risk level: A*+CPSO, A*+CPSO+B-Spline, A*+CPSO+speed adaptation, and the complete method (Table 10). Within this ablation benchmark, all four variants were evaluated using the same seeds and corresponding environmental realizations to ensure paired and internally consistent component-level comparisons. The ablation seed set was generated independently from the seed set used for the main static benchmark reported in Table 4, Table 5 and Table 6. Therefore, the absolute full-method means in Table 10 are not expected to reproduce the values reported in the main static comparison exactly. Because each disabled component is entirely removed from the pipeline rather than merely bypassed, the differences can be read directly as component contributions. B-Spline smoothing accounts for almost all of the roughness reduction: at high risk it lowers the mean roughness from 0.271 rad for the A*+CPSO variant to 0.147 rad, but it simultaneously raises the accumulated dose from 0.148 to 0.171 mSv, because the 1.0 m clearance constraint of the safety-validated smoother pushes the trajectory away from the low-dose shadows located immediately behind the obstacles. Speed adaptation acts in the opposite direction on accumulated dose, reducing the high-risk dose to 0.097 mSv by accelerating through high-dose regions at the cost of a small increase in mission time. The complete method combines these effects, retaining most of the smoothness benefit (0.130 rad) while keeping the dose (0.134 mSv) below that of the smoothing-only variant. This component-level behavior is visualized in Figure 14, where accumulated dose and trajectory roughness are compared across the ablation variants and risk levels. This decomposition shows that the accumulated-dose and smoothness objectives are governed by different components, and that their combination represents a deliberate safety-driven trade-off rather than the effect of a single dominant mechanism.

### 5.4. Sensitivity Analysis

**Figure 15:**
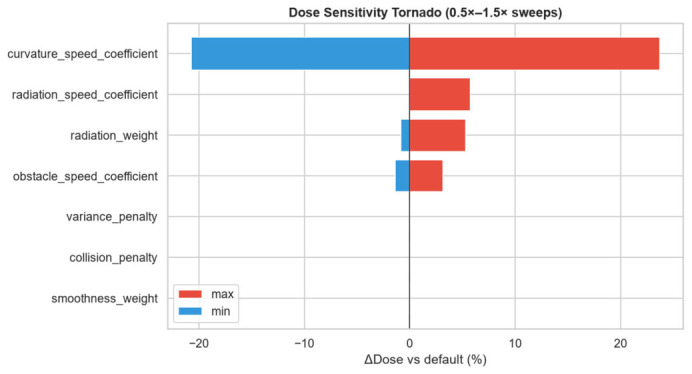
Tornado diagram showing the sensitivity of accumulated dose to a ±50% variation in each cost weight in the medium-risk scenario (30 seeds).

To assess the robustness of the reported behavior to the hand-tuned cost weights, each weight was independently scaled to 0.5, 0.75, 1.0, 1.25, and 1.5 times its default value reported in Table 1 over thirty common seeds, and the resulting change in accumulated dose was measured. Figure 15 summarizes the sensitivity of accumulated dose to these weight variations. The dose is most sensitive to the curvature speed coefficient, which changes it by between −20.7% and +23.7% because it governs how aggressively the robot decelerates in high-curvature maneuvers and, therefore, how long it dwells in exposed regions. The radiation weight and the radiation-speed coefficient have a secondary influence of at most about 5–6%, whereas the collision penalty and the variance penalty behave as near-hard constraints and shift the dose by only a few percent. The absence of any single weight that dominates the dose response indicates that the reported advantage of the proposed method is not an artefact of a particular tuning choice.

### 5.5. Contribution of the Metaheuristic Refinement Under Dynamic Conditions

A recurring concern in the dynamic experiments is whether the CPSO refinement contributes meaningfully once fast reactive replanning is already active. To quantify this effect, every local CPSO activation was logged with its trigger reason, runtime, pre-refinement cost, post-refinement cost, and acceptance status. In addition, the full dynamic pipeline was evaluated with and without the local CPSO refinement stage on matched episodes across the three risk levels.

Across the dynamic metaheuristic variants, CPSO was triggered 5440 times. Among these activations, 1878 produced an accepted update, corresponding to an accepted-improvement rate of 34.5%. The mean runtime per CPSO trigger was 2.83 ms, and the mean runtime of accepted triggers was 3.25 ms, which is well below the predefined 50 ms local CPSO runtime window reported in Table 1.

**Figure 16:**
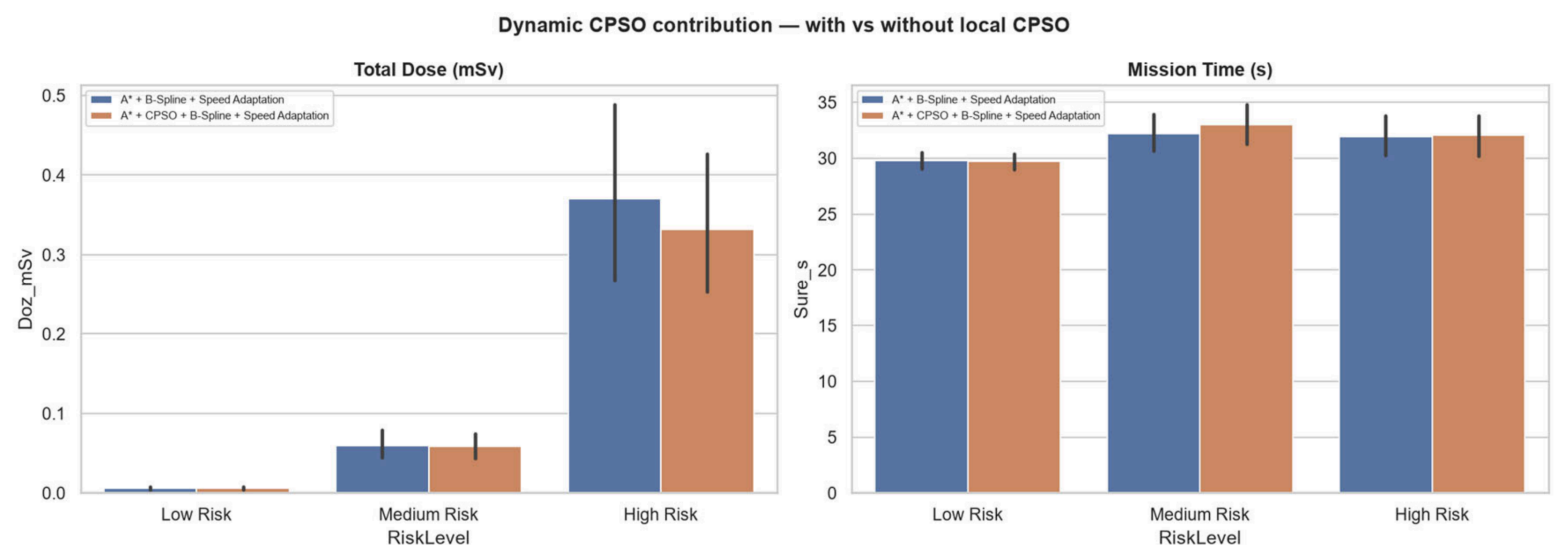
Dynamic contribution of the local CPSO refinement stage. Accumulated dose and mission time are compared for the full pipeline with and without local CPSO refinement on matched dynamic episodes across risk levels.

The paired with/without-CPSO comparison shows that the dynamic contribution of CPSO is risk-dependent. Under low-risk conditions, the dose reduction was negligible (0.2%). Under medium-risk conditions, the dose reduction increased to 2.4%. Under high-risk conditions, where stronger dose-rate gradients and moving-obstacle interactions more frequently invalidate the current route, local CPSO reduced the accumulated dose by 10.4%. This risk-dependent contribution is illustrated in Figure 16, where the accumulated dose and mission time of the full pipeline are compared with and without local CPSO refinement across the three risk levels.

These results indicate that CPSO does not act as a continuously dominant online optimizer in dynamic scenes. Instead, its role is local and event-triggered: it improves the route only when the current path is sufficiently degraded or when a meaningful local refinement opportunity exists. Therefore, its contribution is risk-dependent, and the bounded event-triggered design limits the additional computational burden to cases in which refinement is expected to provide measurable benefit.

### 5.6. Statistical Significance

To evaluate whether the observed performance differences are statistically meaningful, paired statistical tests were performed using matched seeds. Therefore, each compared pair shared the same environment, obstacle trajectories, and measurement-noise realization. The normality of the paired differences was assessed using the Shapiro–Wilk test. If normality was not rejected, a paired *t*-test was used; otherwise, the Wilcoxon signed-rank test was applied. The Holm procedure was used to control the family-wise error rate within each risk-level and metric group. Uncertainty was reported using bootstrap 95% confidence intervals with 5000 resamples, and effect sizes were reported as paired Cohen’s d or rank-biserial correlation, depending on the selected test. For success-rate comparisons, the exact McNemar test was applied using all dynamic episodes, including failed runs. Continuous trajectory metrics, including cumulative dose, path length, mission time, and roughness, were compared only for paired episodes in which both methods produced a valid completed trajectory. Consequently, the number of valid pairs varied across risk levels.

**Figure 17:**
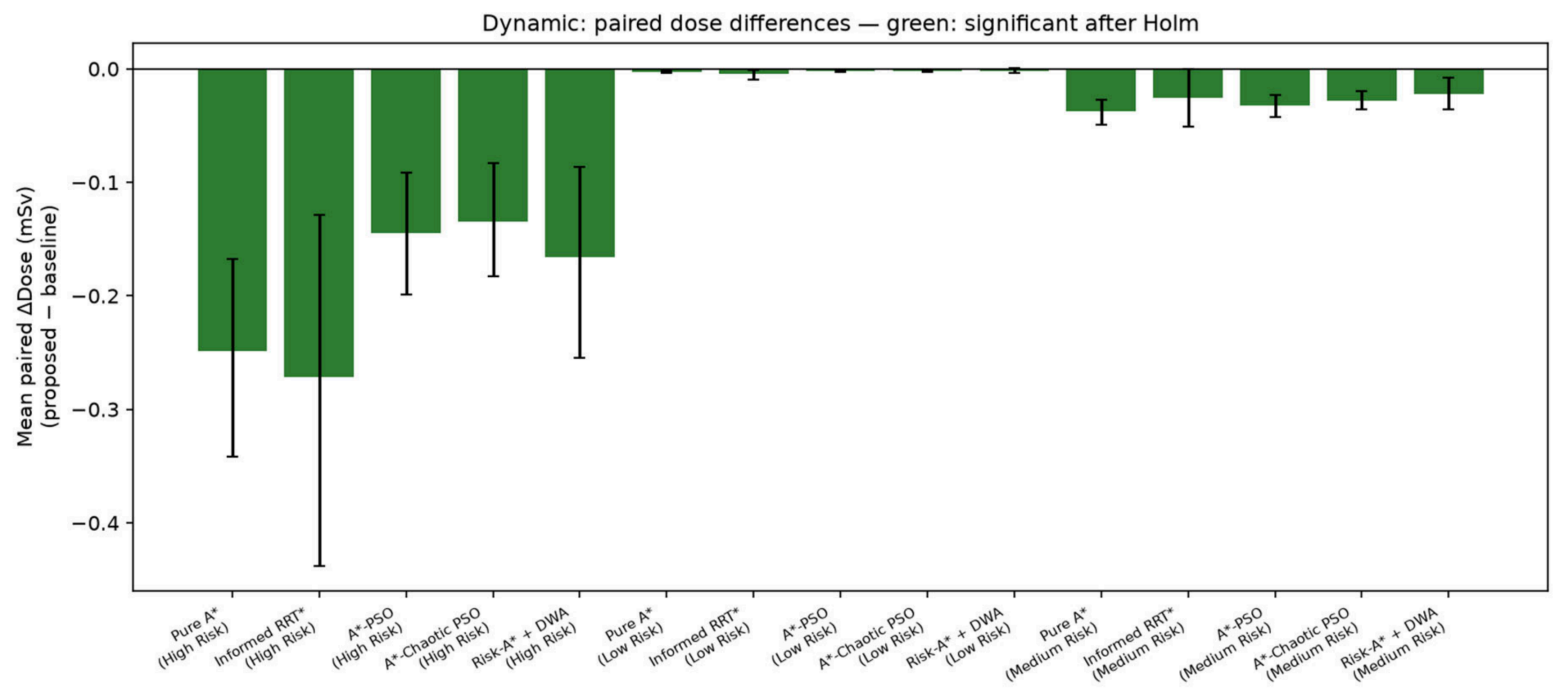
Mean paired difference in accumulated dose between the proposed method and each baseline in the dynamic experiments. Error bars show bootstrap 95% confidence intervals, and comparisons significant after Holm correction are highlighted.

In the dynamic experiments, the accumulated-dose advantage of the proposed method was statistically significant against every baseline at all three risk levels. Overall, 15 out of 15 dynamic dose comparisons were significant after Holm correction, with Holm-adjusted *p* ≤ 0.0006 and effect sizes between 0.45 and 0.96. This paired dose-difference pattern is illustrated in Figure 17, which summarizes the mean paired differences and their bootstrap 95% confidence intervals across the baseline comparisons.

Against Pure A*, the mean paired dose difference was −0.0026 mSv in the low-risk scenario, −0.0373 mSv in the medium-risk scenario, and −0.2490 mSv in the high-risk scenario. The corresponding bootstrap 95% confidence intervals were [−0.0039, −0.0016] mSv, [−0.0492, −0.0269] mSv, and [−0.3420, −0.1675] mSv, respectively. Since all intervals remain below zero, the reduction in accumulated dose is statistically supported despite the relatively large standard deviations in the raw metric tables. The paired comparison against Pure A* is summarized in Table 11.

For success rate, the proposed method was significantly more reliable than Informed RRT* under medium- and high-risk conditions, where the success rates were 89% versus 67% and 89% versus 62%, respectively. In contrast, the success rate of the proposed method was statistically close to the remaining A*-based methods. This indicates that the accumulated-dose reduction was achieved without a corresponding loss in goal-reaching reliability.

These results clarify that the reported dynamic improvements are not only differences in mean values. The paired design removes environment-to-environment variability from the comparison, while the confidence intervals and effect sizes show that the dose reduction is statistically significant and practically meaningful, especially in medium- and high-risk dynamic environments.

Effect sizes are reported as rank-biserial correlations because the Wilcoxon signed-rank test was selected for these paired comparisons.

### 5.7. Computational Performance and Online Execution

Static and dynamic experiments require different computational metrics because static experiments evaluate planning-time cost, whereas dynamic experiments evaluate online execution latency. In the present analysis, static experiments report planning time, whereas dynamic experiments report online execution latency. All experiments were executed on a Windows 10 workstation with a 16-core CPU and 31.7 GB RAM using Python 3.11.7. For each dynamic episode, mean replanning-cycle latency, 95th-percentile step latency, maximum observed step latency, number of replanning calls, replanning runtime, and deadline-miss count were logged. The control period was set to 100 ms, corresponding to the 10 Hz collision-checking and replanning frequency reported in Table 1.

For the proposed method, the equally weighted mean replanning-cycle latency across the low-, medium-, and high-risk scenarios was 2.43 ms. This value was calculated as the arithmetic mean of the three scenario-level means reported in Table 7, Table 8 and Table 9, namely (1.49 + 2.28 + 3.52)/3. When all 26,503 individual replanning calls were pooled, the call-weighted mean latency was 2.65 ms because the number of replanning calls differed across the three risk levels. The mean per-episode 95th-percentile step latency was 9.80 ms, while the mean per-episode maximum latency was 16.46 ms, which remained well below the 100 ms control period. A small number of latency outliers were observed, with an absolute maximum step latency of 315.25 ms across all proposed-method episodes. However, these outliers were rare. Of the 26,503 replanning calls, only 16 exceeded the 100 ms control-period deadline, corresponding to 0.060% of all calls and an average of 0.053 deadline misses per episode. The proposed method required an average of 88.34 replanning calls per episode.

These results indicate that the proposed framework is compatible with online replanning under the tested simulation settings, but they should not be interpreted as a hard real-time guarantee for every robotic platform. Real deployment would require implementation-level timing validation on the target robot hardware and sensor stack. Therefore, this study does not claim hardware-certified real-time performance and instead reports online execution latency relative to the 100 ms simulation control period.

**Figure 18:**
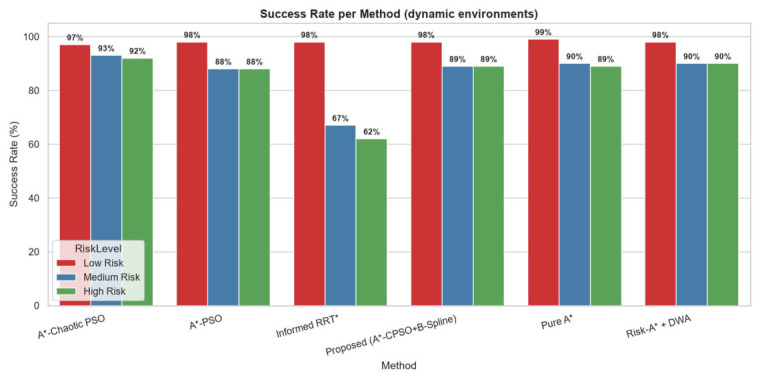
Success rate of each method across the three risk levels in the dynamic experiments.

**Figure 19:**
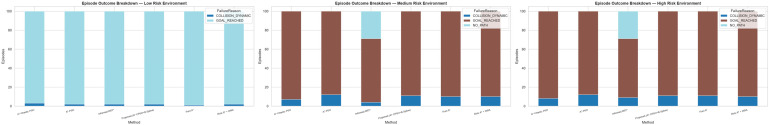
Distribution of the eight episode-outcome types by method for each risk level.

The success-rate results are summarized in Figure 18, which compares the goal-reaching performance of each method across the three dynamic risk levels. The distribution of episode outcomes is shown in Figure 19, including goal reaching, collision-related failures, and no-feasible-path cases. These outcome-level results complement the latency analysis by showing that the proposed method maintains online execution feasibility without sacrificing mission reliability.

The computational complexity of the main components can be summarized as follows. The grid-based A* planner has a complexity of O(E log V), where V and E denote the numbers of grid cells and graph edges, respectively. More precisely, the complexity of the local CPSO stage can be expressed as O(TN C_fit(W)), where T is the number of iterations, N is the swarm size, W is the number of waypoints in the bounded look-ahead window, and C_fit(W) is the cost of evaluating one particle. In the present implementation, the particle-position and velocity updates, path-length calculation, radiation-dose accumulation, smoothness calculation, occupancy and clearance checking, and constraint-penalty evaluation are performed by traversing the local waypoint sequence and a proportional number of segment samples. Radiation, occupancy, and clearance values are obtained through constant-time grid-map lookups. Therefore, C_fit(W) = O(W), and the local CPSO complexity reduces to O(TNW). The comparison and update of the personal and global best solutions introduce an additional O(TN) cost, which is asymptotically dominated by O(TNW). If obstacle clearance were instead calculated by directly comparing every waypoint with M individual obstacle primitives, the corresponding upper bound would become O(TNWM). B-Spline fitting and post-smoothing trajectory validation are performed after CPSO convergence and are therefore accounted for separately as approximately O(Wk^2^) and O(S), respectively, where k is the spline degree, and S is the number of resampled trajectory points used for safety validation. Kalman prediction and update have a complexity of O(B) per control step, where B is the number of moving obstacles, because the state dimension of each obstacle tracker is fixed. Accordingly, when all planning and refinement stages are activated, the dominant complexity of a complete dynamic planning cycle can be represented as O(E log V + TNW + Wk^2^ + S + B) under the grid-lookup assumptions used in this study.

## 6. Conclusions

This study developed a radiation-aware path planning approach for mobile robots operating in static and dynamic task environments containing radiation sources. The proposed architecture integrates A*-based global planning, CPSO-based route refinement, B-Spline trajectory smoothing, and dynamic speed adaptation within the same decision-making process. The radiation field was treated as a physically parameterized dose-rate field that affects both route cost and cumulative dose.

The simulation results showed that, based on the rounded mean values in Table 4, Table 5 and Table 6, the proposed method reduced Total Dose (mSv) by approximately 33.3%, 47.2%, and 21.4% relative to Pure A* under low-, medium-, and high-risk static conditions, respectively. In dynamic scenarios, the corresponding dose reductions were approximately 32.5%, 41.4%, and 40.1%. These findings indicate that the proposed framework consistently reduced cumulative dose relative to Pure A* under both fixed- and moving-obstacle conditions. However, the proposed method did not achieve the lowest static dose among all baselines in the high-risk scenario. The paired statistical analysis further confirmed that the dynamic dose reductions were statistically significant across all baseline comparisons after Holm correction.

In terms of trajectory quality, the ablation results showed that B-Spline smoothing is mainly responsible for roughness reduction, whereas dynamic speed adaptation mainly governs the dose-time trade-off. The local CPSO analysis further showed that CPSO should be interpreted as a bounded, event-triggered refinement component rather than a continuously dominant online optimizer. The latency analysis showed that the proposed method achieved an equally weighted mean replanning-cycle latency of 2.43 ms across the three risk levels and a call-weighted mean latency of 2.65 ms over all 26,503 replanning calls. The mean per-episode 95th-percentile step latency was 9.80 ms under the tested 100 ms simulation control period. However, these results should not be interpreted as a hardware-certified hard real-time guarantee; deployment on a physical robot would require implementation-level timing validation on the target hardware and sensor stack.

Although the model expresses the radiation field in mSv/h and includes line-of-sight shielding attenuation, the present validation remains simulation-based. Therefore, the reported dose values should be interpreted as physically parameterized simulation doses rather than as measurements from a specific nuclear facility. Real-world deployment would require detector calibration, source characterization, shielding verification, and validation against measured dose-rate maps.

## Figures and Tables

**Table 1:** Complete simulation, algorithmic, and physical model parameters used in the experiments.

Group	Parameter	Value
A* cost weights	Astar distance weight	1.0
A* cost weights	Astar radiation weight	0.3
CPSO	Cpso population	20
CPSO	Cpso iterations	60
CPSO	Cpso inertia min	0.35
CPSO	Cpso inertia max	0.85
CPSO	Cpso c1	2.0
CPSO	Cpso c2	2.0
CPSO	Cpso logistic r	3.9
CPSO	Cpso penalty	10,000.0
Local CPSO	Cpso lookahead m	20.0
Local CPSO	Cpso max runtime ms	50.0
Local CPSO	Cpso early stop iters	10
B-Spline	Bspline degree	3
B-Spline	Bspline smoothing	0.0
B-Spline	Bspline samples	150
Safety/replanning	Safety distance m	1.5
Safety/replanning	Goal tolerance m	4.5
Safety/replanning	Replanning threshold m	2.0
Safety/replanning	Collision check hz	10.0
Safety/replanning	Replanning hz	10.0
Speed adaptation	Speed radiation coefficient	1.0
Speed adaptation	Speed curvature coefficient	1.0
Speed adaptation	Speed obstacle coefficient	1.0
Speed adaptation	Min speed m/s	0.8
Speed adaptation	Max speed m/s	6.0
Speed adaptation	Nominal speed m/s	3.0
Speed adaptation	Max accel m/s^2^	2.0
Speed adaptation	Speed dose reference msv/h	1.0
Radiation physics	Grid Cell size m	1.0
Radiation physics	radiation_r0_m	1.0
Radiation physics	radiation_rmin_m	0.5
Radiation physics	material_mu_air_per_m	0.01
Radiation physics	material_mu_concrete_per_m	14.0
Radiation physics	material_mu_steel_per_m	42.0
Radiation physics	material_mu_lead_per_m	66.0
Radiation physics	dose_calibration_msv_h_per_unit	0.01
Dose thresholds	max_total_dose_msv	50.0
Dose thresholds	max_dose_rate_msv_h	100.0
Variance safety cost	safety_occupancy_weight	5.0
Variance safety cost	safety_variance_weight	1.0
Variance safety cost	safety_narrow_weight	2.0
Variance safety cost	safety_k_sigma	1.0
Kalman tracker	kalman_measurement_noise_std	0.5
Kalman tracker	kalman_process_noise_std	0.1
Kalman tracker	kalman_prediction_horizon_s	3.0
Common budget	budget_max_wall_time_ms	500.0
Common budget	budget_max_iterations	60
Common budget	budget_control_period_ms	100.0

**Table 2:** Risk profiles used in static and dynamic simulations with physically parameterized source dose rates and shielding materials.

Risk Level	Number of Sources	Source Dose $D_{0}$ (mSv/h)	Shielding
Low	3–6	30–60	concrete, $\mu =14\;m^{-1}$
Medium	8–12	100–200	concrete/steel
High	18–25	400–600	concrete/steel/lead

**Table 3:** Performance metrics used in the experimental evaluation.

Metric	Definition	Preferred Direction
Total Dose (mSv)	Cumulative ABSOLUTE absorbed dose along the trajectory: Σ dose_rate(mSv/h)·dt/3600, using the actual velocity profile. Lower is better.	Lower is better
Path Length	Total Euclidean path length from start to goal, in meters.	Lower is better
Time	Total travel time based on the velocity profile.	Lower is better
Roughness	Average absolute heading change between consecutive route or trajectory segments, in radians.	Lower is better
Computational Time/Step Latency	Per-stage planning wall-time (static) and per-step latency mean/p95/max (dynamic), in ms. Lower is better.	Lower is better
Conv	Euclidean distance between the final robot position and the goal center, in meters.	Lower is better
Success Rate	Successful mission completion rate.	Higher is better

**Table 4:** Low-risk metric comparison for static environment (mean ± std).

Method	Total Dose (mSv)	Path Length (m)	Time (s)	Roughness (rad)	Planning Time (ms)
Pure A*	0.009 ± 0.019	109.880 ± 6.070	36.627 ± 2.023	0.197 ± 0.061	133.932 ± 315.866
Informed RRT*	0.006 ± 0.013	112.898 ± 9.528	37.633 ± 3.176	0.319 ± 0.077	500.582 ± 0.422
A*-PSO	0.006 ± 0.011	109.727 ± 5.335	36.576 ± 1.778	0.406 ± 0.052	144.893 ± 328.781
A*-CPSO	0.008 ± 0.015	109.487 ± 5.402	36.496 ± 1.801	0.389 ± 0.055	148.143 ± 331.590
Risk-A*+DWA	0.008 ± 0.023	110.666 ± 5.488	36.211 ± 3.288	0.164 ± 0.041	145.577 ± 315.568
Proposed	0.006 ± 0.010	104.410 ± 6.541	34.803 ± 2.180	0.059 ± 0.014	142.981 ± 20.516

**Table 5:** Medium-risk metric comparison for static environment (mean ± std).

Method	Total Dose (mSv)	Path Length (m)	Time (s)	Roughness (rad)	Planning Time (ms)
Pure A*	0.072 ± 0.154	134.119 ± 7.530	44.706 ± 2.510	0.096 ± 0.074	58.571 ± 72.519
Informed RRT*	0.042 ± 0.064	133.392 ± 9.498	44.464 ± 3.166	0.267 ± 0.078	500.514 ± 0.481
A*-PSO	0.056 ± 0.123	134.046 ± 7.985	44.682 ± 2.662	0.346 ± 0.072	67.628 ± 78.696
A*-CPSO	0.051 ± 0.102	133.674 ± 7.725	44.558 ± 2.575	0.322 ± 0.057	70.020 ± 77.498
Risk-A*+DWA	0.044 ± 0.081	134.564 ± 7.569	44.539 ± 4.220	0.148 ± 0.054	69.852 ± 71.551
Proposed	0.038 ± 0.076	131.856 ± 27.707	43.952 ± 9.236	0.062 ± 0.037	137.306 ± 59.555

**Table 6:** High-risk metric comparison for static environment (mean ± std).

Method	Total Dose (mSv)	Path Length (m)	Time (s)	Roughness (rad)	Planning Time (ms)
Pure A*	0.201 ± 0.418	137.764 ± 1.408	45.921 ± 0.469	0.019 ± 0.016	12.020 ± 10.013
Informed RRT*	0.251 ± 0.438	138.435 ± 1.354	46.145 ± 0.451	0.115 ± 0.029	500.639 ± 0.707
A*-PSO	0.126 ± 0.239	139.009 ± 1.819	46.336 ± 0.606	0.277 ± 0.050	20.261 ± 9.809
A*-CPSO	0.137 ± 0.274	138.905 ± 1.820	46.302 ± 0.607	0.265 ± 0.037	23.720 ± 9.954
Risk-A*+DWA	0.126 ± 0.268	139.471 ± 1.818	46.381 ± 3.815	0.134 ± 0.061	24.639 ± 9.930
Proposed	0.158 ± 0.347	134.184 ± 17.230	44.728 ± 5.743	0.056 ± 0.027	124.629 ± 16.287

**Table 7:** Low-risk metric comparison for dynamic environment (mean ± std).

Method	Total Dose (mSv)	Path Length (m)	Time (s)	Roughness (rad)	Replanning-Cycle Latency (ms)	Success (%)
Pure A*	0.0083 ± 0.0143	99.3 ± 6.6	33.1 ± 2.2	0.194 ± 0.067	1.28 ± 0.27	99%
Informed RRT*	0.0102 ± 0.0232	100.2 ± 6.6	33.4 ± 2.2	0.207 ± 0.077	2.71 ± 1.94	98%
A*-PSO	0.0073 ± 0.0112	99.0 ± 5.6	33.0 ± 1.9	0.197 ± 0.075	1.40 ± 0.35	98%
A*-CPSO	0.0075 ± 0.0114	98.9 ± 6.0	33.0 ± 2.0	0.193 ± 0.068	1.40 ± 0.19	97%
Risk-A*+DWA	0.0073 ± 0.0120	99.1 ± 5.5	33.0 ± 1.8	0.173 ± 0.056	1.25 ± 0.28	98%
Proposed	0.0056 ± 0.0106	99.6 ± 6.5	29.9 ± 3.9	0.152 ± 0.060	1.49 ± 0.29	98%

**Table 8:** Medium-risk metric comparison for dynamic environment (mean ± std).

Method	Total Dose (mSv)	Path Length (m)	Time (s)	Roughness (rad)	Replanning-Cycle Latency (ms)	Success (%)
Pure A*	0.0941 ± 0.1222	113.4 ± 14.7	37.8 ± 4.9	0.265 ± 0.108	2.15 ± 1.54	90%
Informed RRT*	0.0812 ± 0.0986	112.0 ± 10.9	37.3 ± 3.6	0.236 ± 0.086	5.66 ± 9.30	67%
A*-PSO	0.0858 ± 0.1052	113.3 ± 15.9	37.8 ± 5.3	0.264 ± 0.101	2.22 ± 1.45	88%
A*-CPSO	0.0815 ± 0.0932	115.2 ± 16.2	38.4 ± 5.4	0.280 ± 0.115	2.77 ± 3.32	93%
Risk-A*+DWA	0.0761 ± 0.0938	115.1 ± 16.6	38.4 ± 5.5	0.250 ± 0.109	2.34 ± 2.16	90%
Proposed	0.0551 ± 0.0754	113.7 ± 16.7	32.5 ± 8.9	0.229 ± 0.107	2.28 ± 1.21	89%

**Table 9:** High-risk metric comparison for dynamic environment (mean ± std).

Method	Total Dose (mSv)	Path Length (m)	Time (s)	Roughness (rad)	Replanning-Cycle Latency (ms)	Success (%)
Pure A*	0.5890 ± 0.7787	118.1 ± 18.5	39.4 ± 6.2	0.265 ± 0.122	3.50 ± 3.91	89%
Informed RRT*	0.6317 ± 0.7625	120.0 ± 22.7	40.0 ± 7.6	0.235 ± 0.079	5.74 ± 3.35	62%
A*-PSO	0.4745 ± 0.5354	119.3 ± 18.7	39.8 ± 6.2	0.276 ± 0.135	3.92 ± 6.22	88%
A*-CPSO	0.4829 ± 0.5311	117.7 ± 17.9	39.2 ± 6.0	0.278 ± 0.128	3.69 ± 4.55	92%
Risk-A*+DWA	0.5091 ± 0.5959	118.0 ± 18.7	39.3 ± 6.2	0.231 ± 0.123	3.34 ± 3.54	90%
Proposed	0.3526 ± 0.4344	118.6 ± 18.2	32.5 ± 9.2	0.237 ± 0.109	3.52 ± 3.01	89%

**Table 10:** Ablation study: mean +/− standard deviation of accumulated dose and trajectory roughness for the four component variants across the three risk levels (100 seeds each).

Risk Level	Variant	Total Dose (mSv)	Roughness (rad)
Low Risk	A* + CPSO	0.0077 ± 0.0127	0.398 ± 0.056
Low Risk	A* + CPSO + B-Spline	0.0087 ± 0.0185	0.166 ± 0.036
Low Risk	A* + CPSO + Speed Adaptation	0.0060 ± 0.0097	0.392 ± 0.058
Low Risk	A* + CPSO + B-Spline + Speed Adaptation	0.0069 ± 0.0176	0.171 ± 0.058
Medium Risk	A* + CPSO	0.0519 ± 0.0969	0.327 ± 0.067
Medium Risk	A* + CPSO + B-Spline	0.0706 ± 0.1501	0.150 ± 0.059
Medium Risk	A* + CPSO + Speed Adaptation	0.0497 ± 0.1190	0.323 ± 0.071
Medium Risk	A* + CPSO + B-Spline + Speed Adaptation	0.0426 ± 0.0829	0.160 ± 0.075
High Risk	A* + CPSO	0.1479 ± 0.3788	0.271 ± 0.047
High Risk	A* + CPSO + B-Spline	0.1707 ± 0.3411	0.147 ± 0.070
High Risk	A* + CPSO + Speed Adaptation	0.0968 ± 0.2002	0.264 ± 0.042
High Risk	A* + CPSO + B-Spline + Speed Adaptation	0.1342 ± 0.2672	0.130 ± 0.061

**Table 11:** Paired statistical comparison between the proposed method and Pure A* for dynamic Total Dose.

Risk Level	*n* Pairs	Mean Paired ΔDose (mSv)	95% CI (mSv)	Holm-Adjusted *p*	Effect Size
Low Risk	98	−0.0026	[−0.0039, −0.0016]	<0.0001	0.88
Medium Risk	85	−0.0373	[−0.0492, −0.0269]	<0.0001	0.96
High Risk	82	−0.2490	[−0.3420, −0.1675]	<0.0001	0.94

## Data Availability

The datasets generated and/or analyzed during the current study are available from the corresponding author on reasonable request.
